# Supplementary material for: Highly host-linked viromes in the built environment possess habitat-dependent diversity and functions for potential virus-host coevolution
Source: Nat Commun. 2023 May 9;14:2676. doi: 10.1038/s41467-023-38400-0 (PMC10169181; doi:10.1038/s41467-023-38400-0)
Supplement: Supplementary file 1 — Supplementary information [file 41467_2023_38400_MOESM1_ESM.pdf]

Supplementary Figures

Highly host-linked viromes in the built environment possess habitat-dependent diversity and functions for potential virus-host coevolution

Shicong Du,[1] Xinzhao Tong,[1,2] Alvin C. K. Lai,[1] Chak K. Chan,[1] Christopher E. Mason,[3,4,5,6] and Patrick K. H. Lee[1,7]*

[1]School of Energy and Environment, City University of Hong Kong, Hong Kong SAR, China

[2]Department of Biological Sciences, School of Science, Xi'an Jiaotong-Liverpool University, Suzhou, P. R. China

[3]Department of Physiology and Biophysics, Weill Cornell Medicine, New York, NY, USA

[4]The HRH Prince Alwaleed Bin Talal Bin Abdulaziz Alsaud Institute for Computational Biomedicine, Weill Cornell Medicine, New York, NY, USA

[5]The WorldQuant Initiative for Quantitative Prediction, Weill Cornell Medicine, New York, NY, USA

[6]The Feil Family Brain and Mind Research Institute, Weill Cornell Medicine, New York, NY, USA

[7]State Key Laboratory of Marine Pollution, City University of Hong Kong, Hong Kong SAR, China

Correspondence: *B5423, Yeung Kin Man Academic Building, School of Energy and Environment, City University of Hong Kong, Tat Chee Avenue, Kowloon, Hong Kong SAR, China; E-mail: patrick.kh.lee@cityu.edu.hk; Tel: (852) 3442-4625; Fax: (852) 3442-0688.

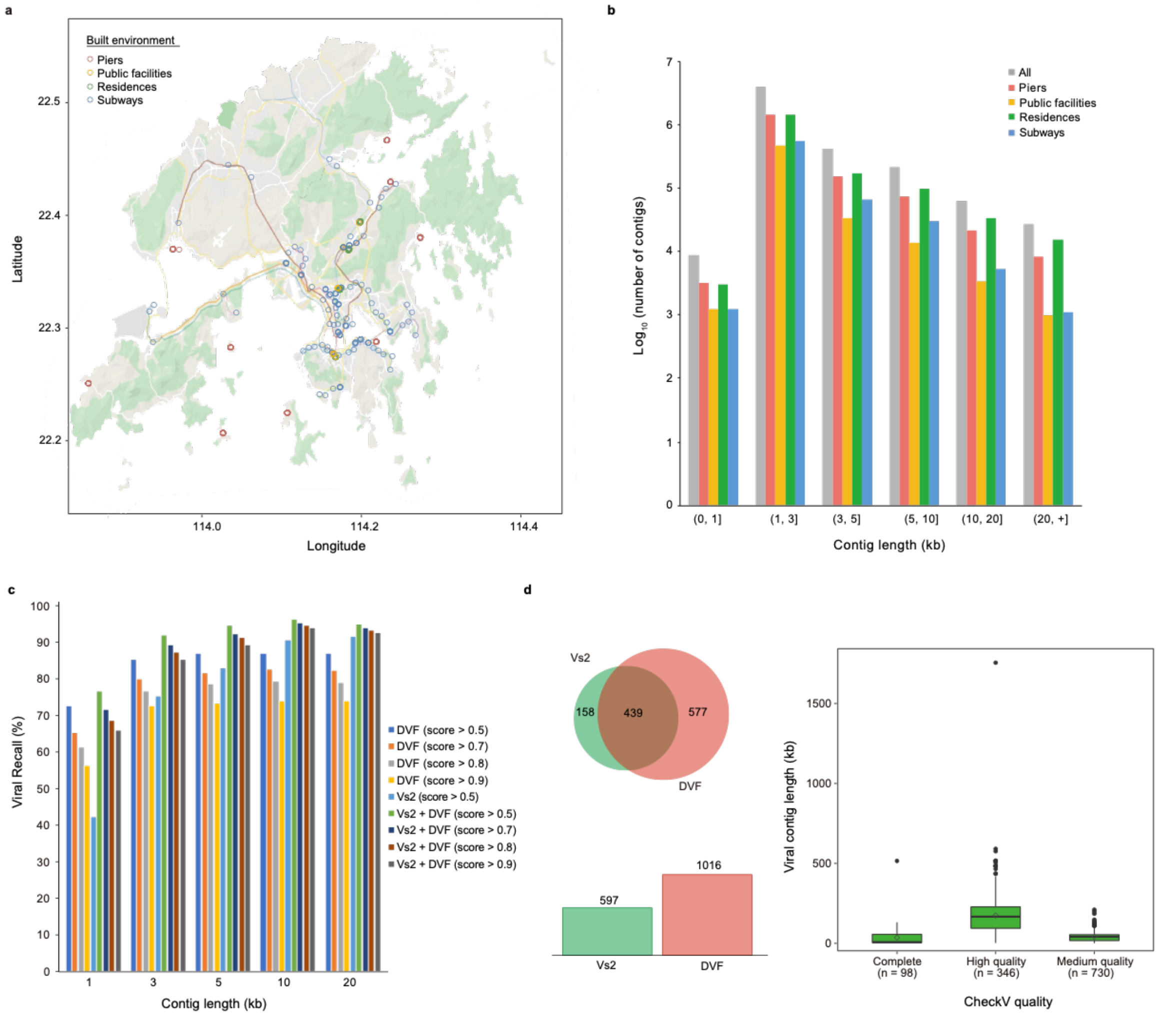

Fig. S1 An overview of the built environment (BE) metagenomes. **(a)** Locations at which the BE metagenome samples were collected in Hong Kong. A freely available map (https://github.com/Paulkit/HKMap) was used as the background image. **(b)** Frequency histogram of the assembled contig lengths from the BE metagenomes. **(c)** The viral recall of mock data using Virsorter2 (Vs2) and DeepVirFinder (DVF). **(d)** Comparison of the number of high-quality viral genomes identified by Vs2 and DVF (left panel), and boxplots of the viral contig lengths of the final set of high-quality viral genomes obtained from the BE metagenomes (right panel). Box plots represent the median, the first quartiles and third quartiles with whiskers drawn within the 1.5 interquartile range value whereas the diamonds represent the mean value. Points outside the whiskers are outliers.

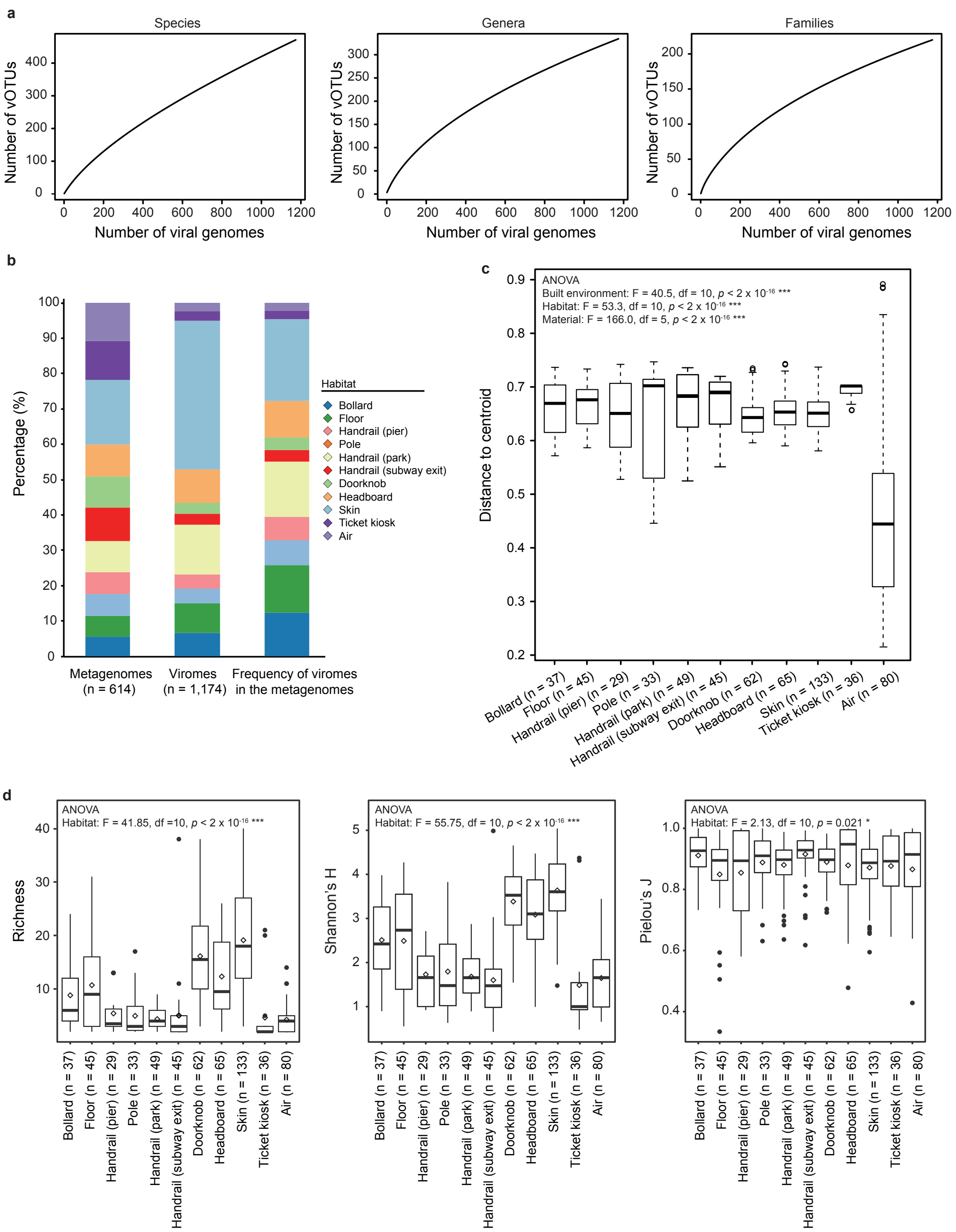

Fig. S2 Diversity of the built environment (BE) viromes. **(a)** Accumulation curves of the viral genomes clustered at the species, genus, and family levels. **(b)** The distribution of metagenomes or viromes in the BE habitats. The frequency of viromes in the metagenomes is calculated by dividing the number of identified viruses by the number of metagenomes in each habitat. **(c)** Boxplots of the distance to centroid of the permutational analysis of multivariate dispersions (PERMDISP) for each habitat. *** denotes $p < 0.001$ according to one-way ANOVA. **(d)** Boxplots of the alpha diversity indexes of the BE viromes, namely richness, Shannon's H, and Pielou's J evenness. * denotes $p < 0.05$ and *** denotes $p < 0.001$ according to one-way ANOVA. Box plots represent the median, the first quartiles and third quartiles with whiskers drawn within the 1.5 interquartile range value whereas the diamonds represent the mean value. Points outside the whiskers are outliers.

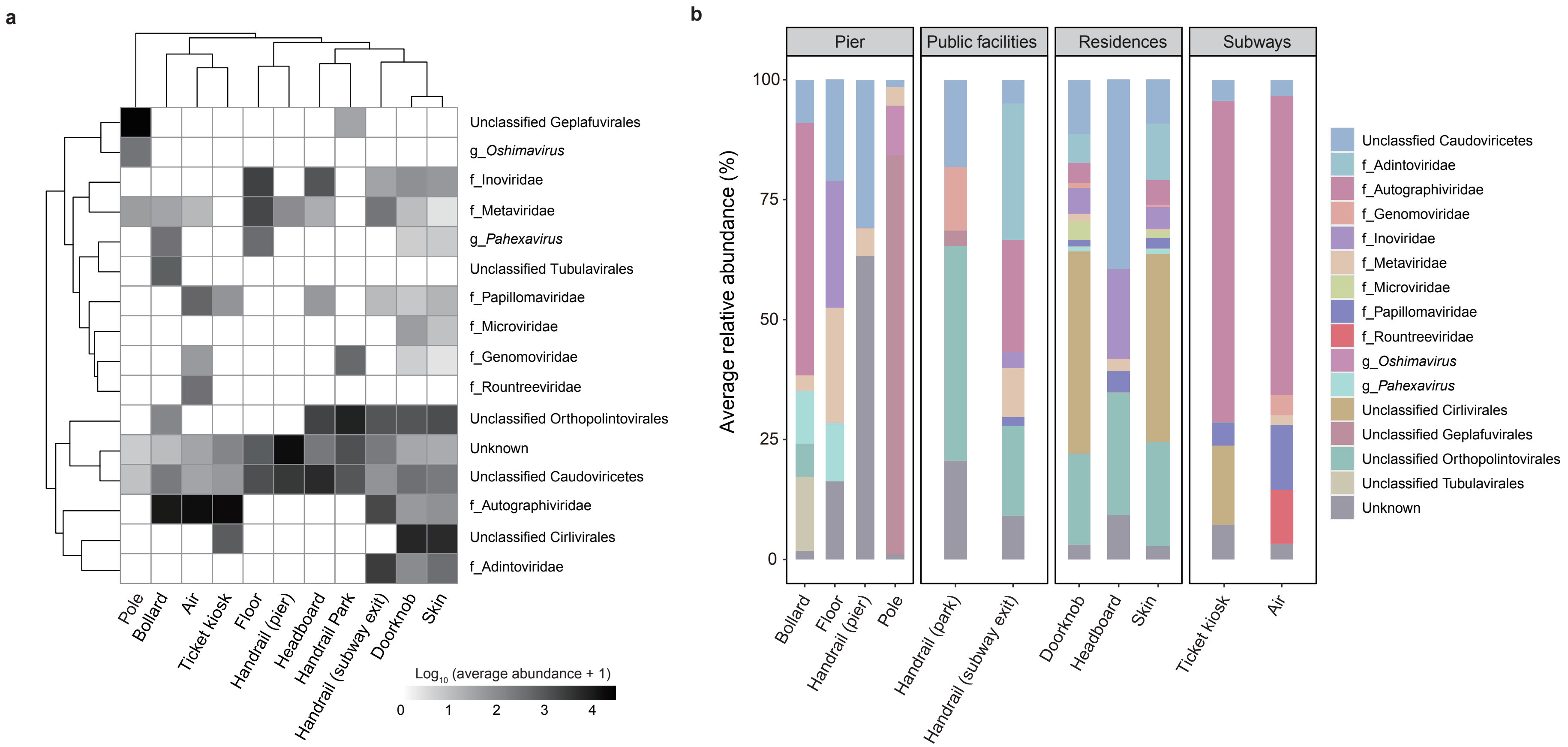

Fig. S3 Taxonomic distribution of the viral operational taxonomic units (vOTUs) across all habitats. **(a)** Heatmap of the average abundance of the vOTUs across the built environment (BE) habitats. **(b)** Barplots of the average relative abundance of the vOTUs across the BE habitats.

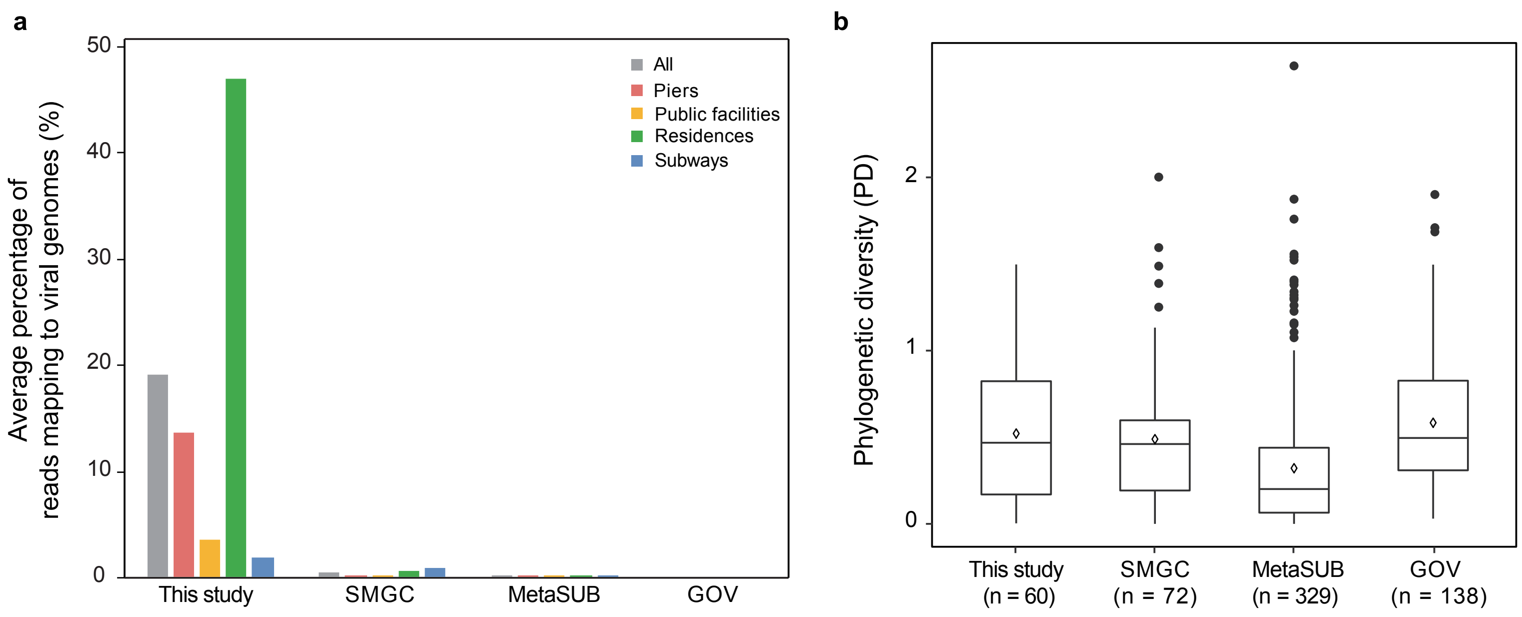

Fig. S4 Comparison of viral operational taxonomic units (vOTUs) from this study and other datasets (SMGC, GOV, and MetaSUB). **(a)** Average percentages of reads from this study that can be mapped to the viral genomes of the four datasets. **(b)** Phylogenetic diversity (PD) of the vOTUs in each dataset. PD was calculated by summing the branch lengths represented by the vOTUs. Box plots represent the median, the first quartiles and third quartiles with whiskers drawn within the 1.5 interquartile range value whereas the diamonds represent the mean value. Points outside the whiskers are outliers.

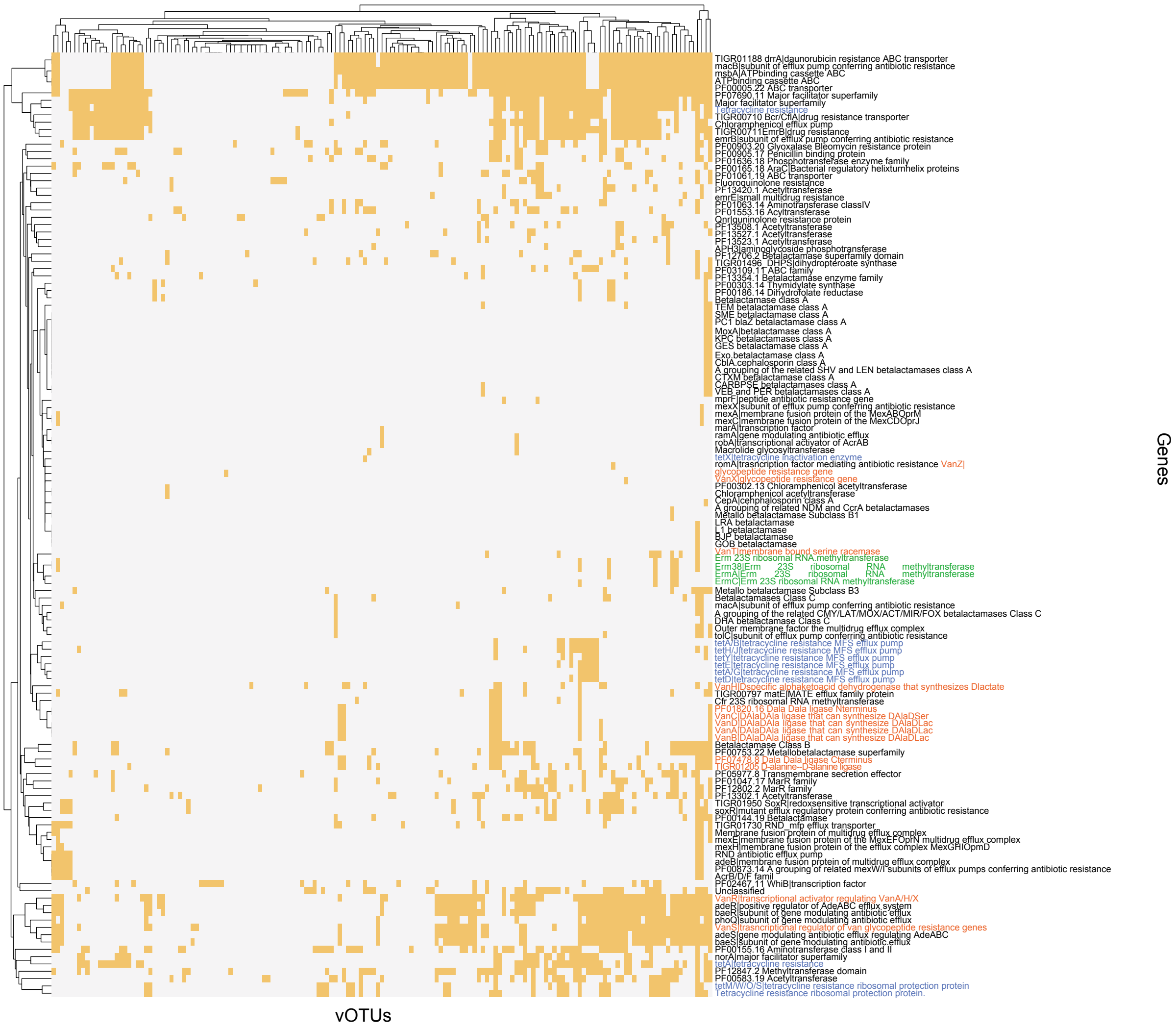

Fig. S5 Heatmap of the antimicrobial resistance genes (ARGs) identified in each viral operational taxonomic unit (vOTU) according to Resfams. Genes that are present (sand yellow) or absent (light gray) are indicated. Hierarchical clustering was used to cluster the ARGs (rows) and vOTUs (columns). Genes conferring resistance to macrolides, vancomycin, and tetracycline are highlighted in green, orange, and blue, respectively.

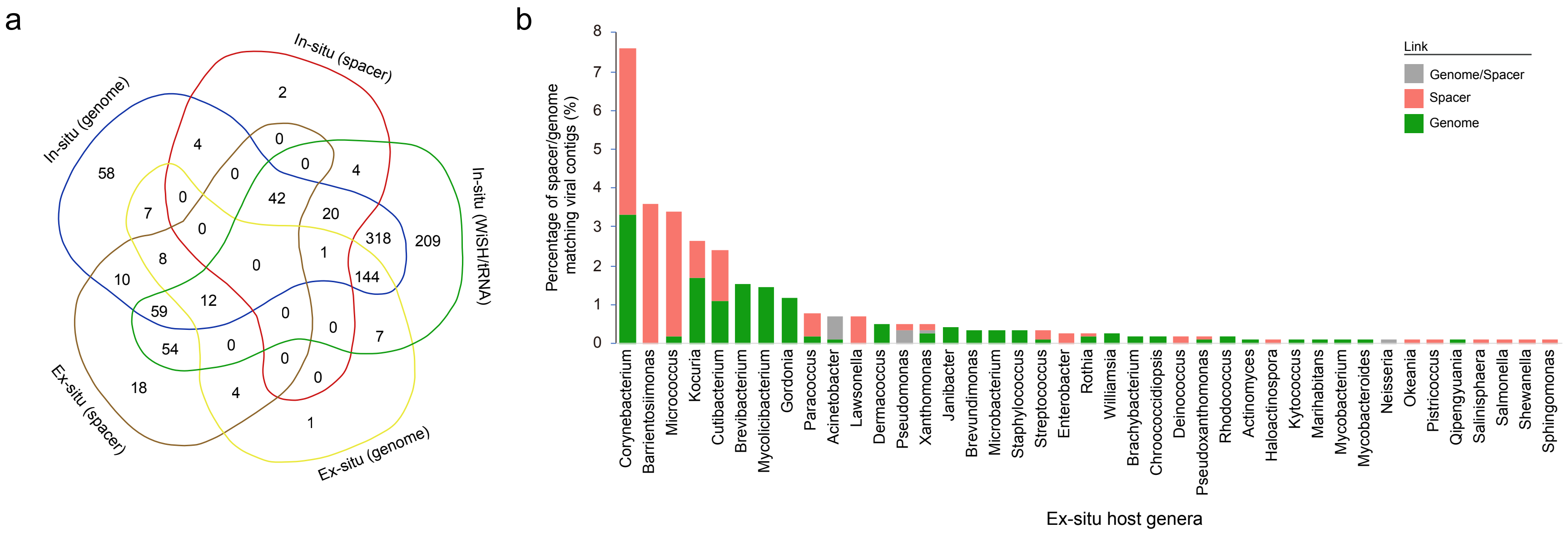

Fig. S6 Virus–host links in the built environments (BEs) based on ex-situ and in-situ host prediction methods. **(a)** A Venn diagram of the number of matches identified using the ex-situ and in-situ host prediction methods. **(b)** The predicted ex-situ hosts for the BE viral genomes. The colors of the bars indicate the various prediction methods

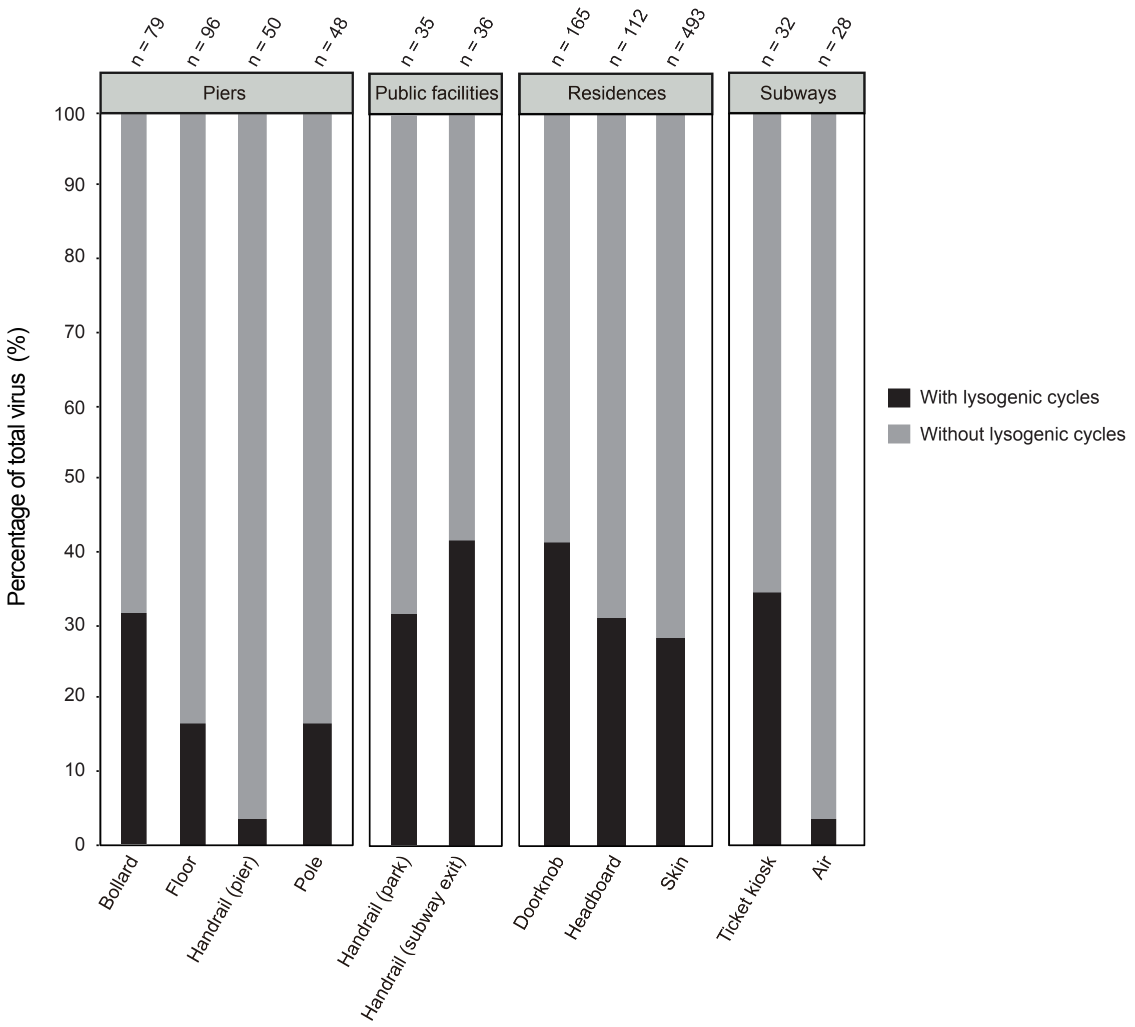

Fig. S7 The proportions of viruses with lysogenic and non-lysogenic cycles in each built environment habitat. The total number of viral genomes in each habitat is indicated at the top.

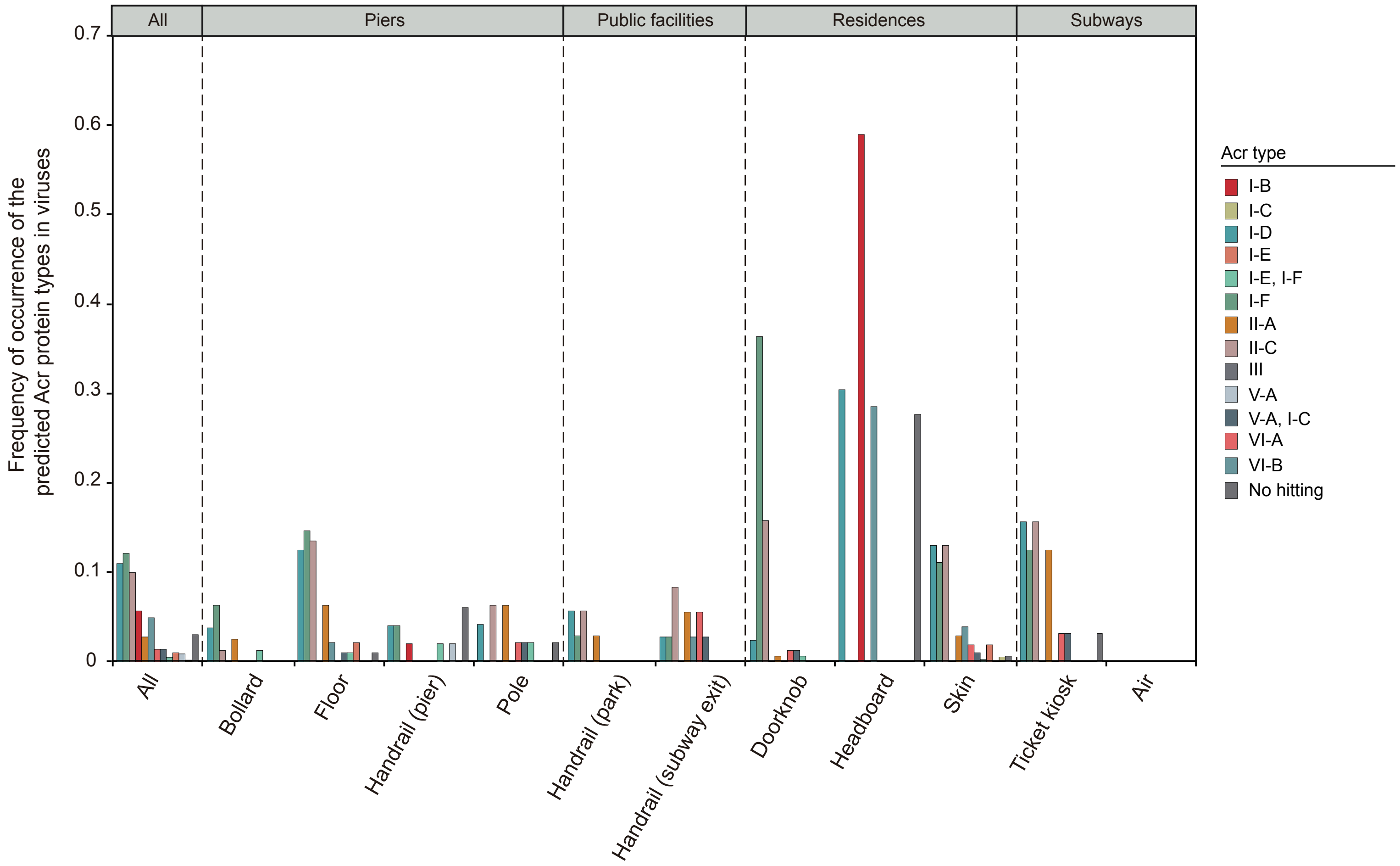

Fig. S8 Frequency of the occurrence of different types of predicted anti-CRISPR (Acr) proteins in each built environment habitat.

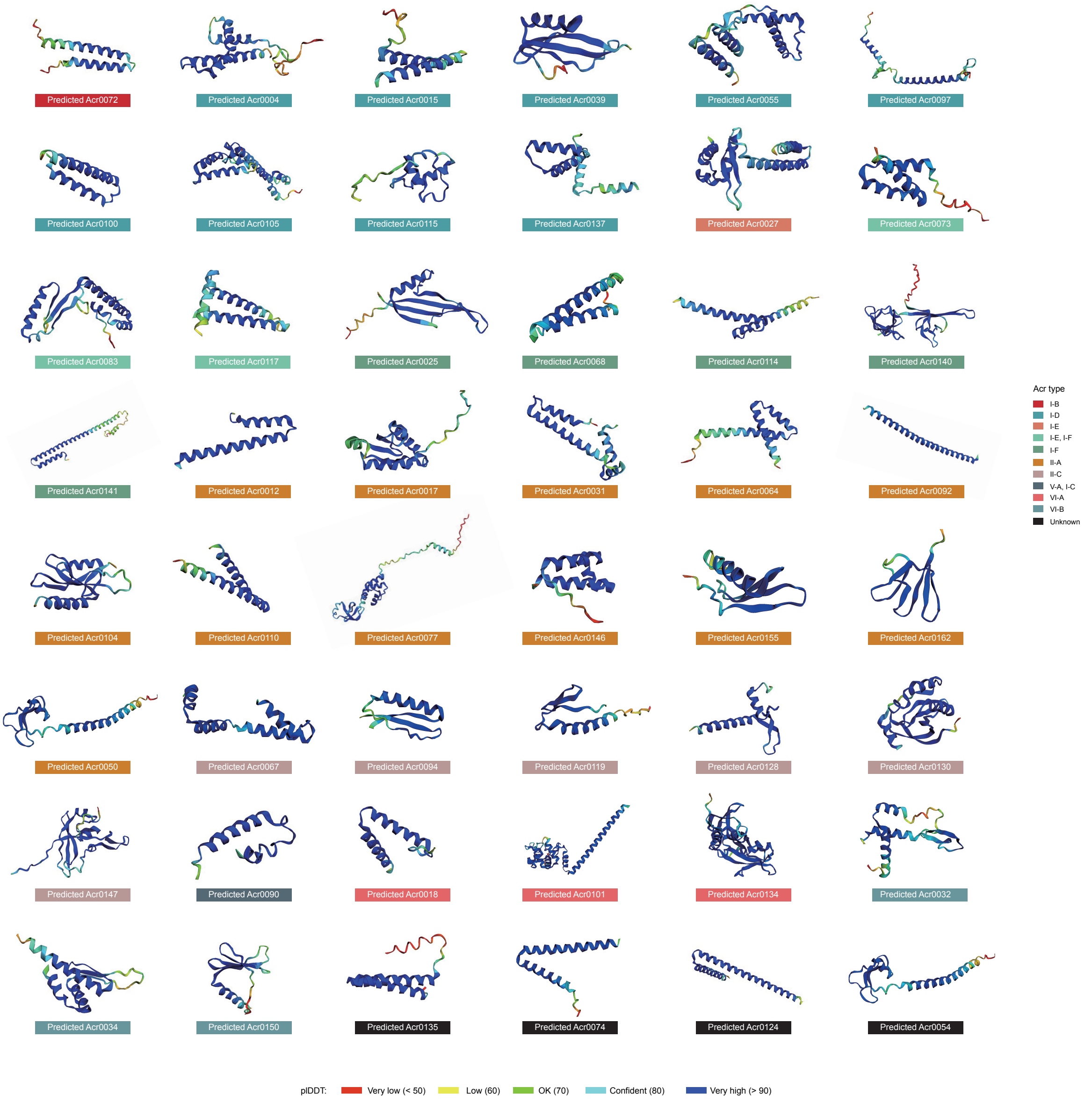

Fig. S9 Structures of the predicted Acr proteins identified in this study. Structure prediction was performed using AlphaFold2 and only the 48 high confidence ones (plDDT > 80) are shown (arranged according to Acr type).

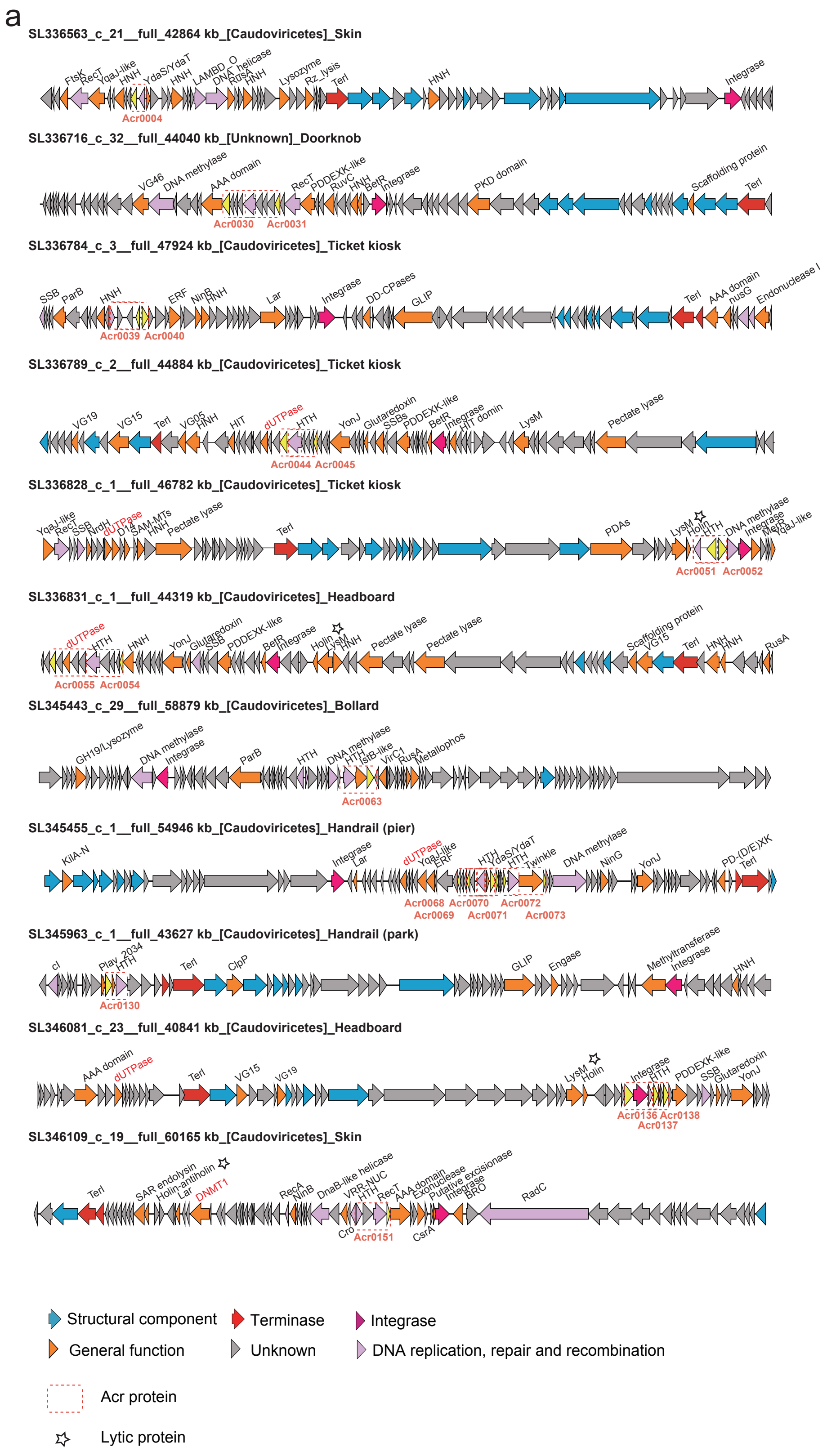
a
SL336563_c_21__full_42864 kb_[Caudoviricetes]_Skin
Acr0004
SL336716_c_32__full_44040 kb_[Unknown]_Doorknob
Acr0030 Acr0031
SL336784_c_3__full_47924 kb_[Caudoviricetes]_Ticket kiosk
Acr0039 Acr0040
SL336789_c_2__full_44884 kb_[Caudoviricetes]_Ticket kiosk
Acr0044 Acr0045
SL336828_c_1__full_46782 kb_[Caudoviricetes]_Ticket kiosk
Acr0051 Acr0052
SL336831_c_1__full_44319 kb_[Caudoviricetes]_Headboard
Acr0055 Acr0054
SL345443_c_29__full_58879 kb_[Caudoviricetes]_Bollard
Acr0063
SL345455_c_1__full_54946 kb_[Caudoviricetes]_Handrail (pier)
Acr0068 Acr0070 Acr0072
Acr0069 Acr0071 Acr0073
SL345963_c_1__full_43627 kb_[Caudoviricetes]_Handrail (park)
Acr0130
SL346081_c_23__full_40841 kb_[Caudoviricetes]_Headboard
Acr0136 Acr0138
Acr0137
SL346109_c_19__full_60165 kb_[Caudoviricetes]_Skin
Acr0151
Structural component
Terminase
Integrase
General function
Unknown
DNA replication, repair and recombination
Acr protein
Lytic protein

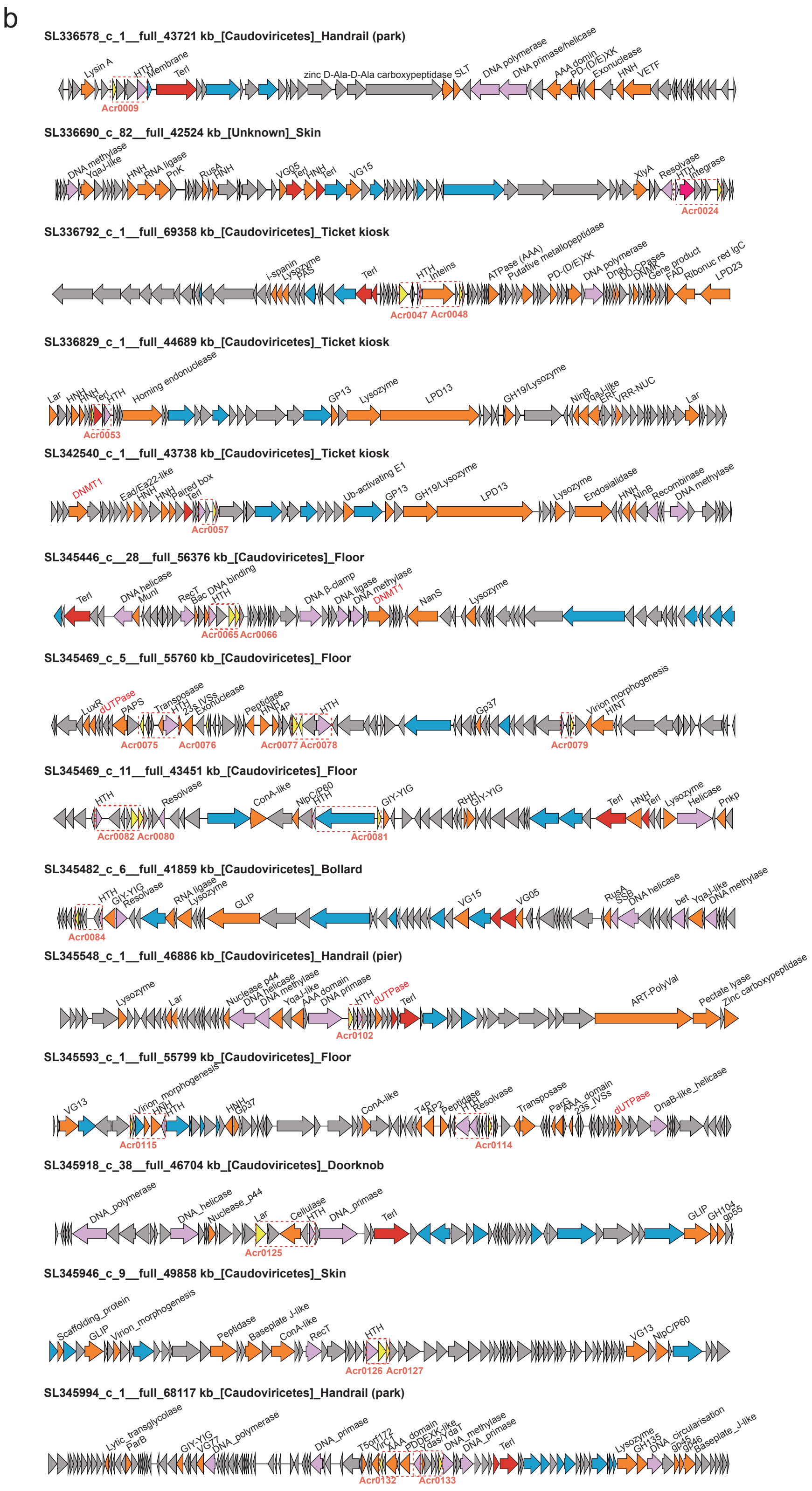

Fig. S10 Functional annotation of all the complete circular viral operational taxonomic units (vOTUs) found to make the **(a)** lysogenic or **(b)** lytic cycle in the built environments. The anti-CRISPR (Acr) protein is highlighted by an orange dashed box, and the auxiliary metabolic gene (AMG) is highlighted in green.
