# Supplementary material for: Highly host-linked viromes in the built environment possess habitat-dependent diversity and functions for potential virus-host coevolution
Source: Nat Commun. 2023 May 9;14:2676. doi: 10.1038/s41467-023-38400-0 (PMC10169181; doi:10.1038/s41467-023-38400-0)
Supplement: Supplementary file 3 — Description of additional supplementary files [file 41467_2023_38400_MOESM3_ESM.docx]

**Description of additional supplementary files**

**Highly host-linked viromes in the built environment possess habitat-dependent diversity and functions for potential virus-host coevolution**

Shicong Du,^1^ Xinzhao Tong,^1, 2^ Alvin C. K. Lai,^1^ Chak K. Chan,^1^ Christopher E. Mason,^3,4,5,6^ and Patrick K. H. Lee^1,7^*

^1^School of Energy and Environment, City University of Hong Kong, Hong Kong SAR, China

^2^Department of Biological Sciences, School of Science, Xi’an Jiaotong-Liverpool University, Suzhou, P. R. China

^3^Department of Physiology and Biophysics, Weill Cornell Medicine, New York, NY, USA

^4^The HRH Prince Alwaleed Bin Talal Bin Abdulaziz Alsaud Institute for Computational Biomedicine, Weill Cornell Medicine, New York, NY, USA

^5^The WorldQuant Initiative for Quantitative Prediction, Weill Cornell Medicine, New York, NY, USA

^6^The Feil Family Brain and Mind Research Institute, Weill Cornell Medicine, New York, NY, USA

^7^State Key Laboratory of Marine Pollution, City University of Hong Kong, Hong Kong SAR, China

**Correspondence**: *B5423, Yeung Kin Man Academic Building, School of Energy and Environment, City University of Hong Kong, Tat Chee Avenue, Kowloon, Hong Kong SAR, China; E-mail: patrick.kh.lee@cityu.edu.hk; Tel: (852) 3442-4625; Fax: (852) 3442-0688.

Supplementary Data 1. Sample information and metadata.

Supplementary Data 2. Information about the 1,174 high-quality viral genomes.

Supplementary Data 3. Alpha diversity of viral operational taxonomic units (vOTUs) across habitats in the built environments.

Supplementary Data 4. Taxonomy of vOTUs across habitats in the built environments.

Supplementary Data 5. Information of the 860 representative metagenome-assembled genomes.

Supplementary Data 6. Predicted ex-situ hosts of the viral genomes in the built environments.

Supplementary Data 7. Predicted in-situ hosts of the viral genomes in the built environments.

Supplementary Data 8. Pearson’s correlations between the normalized abundances of viruses and predicted hosts (n = 614 samples).

Supplementary Data 9. Properties of the CRISPR-Cas systems in the 860 representative metagenome-assembled genomes (rMAGs).

Supplementary Data 10. Properties of the predicted anti-CRISPR (Acr) proteins in the built environment viromes.

Supplementary Data 11. Properties of the predicted auxiliary metabolic genes (AMGs) in the built environment viromes.
